# *Neisseria gonorrhoeae* Resistant to Ceftriaxone and Cefixime, Argentina

**DOI:** 10.3201/eid2206.152091

**Published:** 2016-06

**Authors:** Ricardo Gianecini, Claudia Oviedo, Graciela Stafforini, Patricia Galarza

**Affiliations:** Instituto Nacional de Enfermedades Infecciosas–Administracion Nacional de Laboratorios E Institutos de Salud Dr. Carlos G. Malbran, Buenos Aires, Argentina (R. Gianecini, C. Oviedo, P. Galarza);; Hospital Interzonal Artémides Zatti, Rio Negro, Argentina (G. Stafforini)

**Keywords:** Neisseria gonorrhoeae, bacteria, ceftriaxone, cefixime, antimicrobial resistance, Argentina

**To the Editor:** Antimicrobial resistance in *Neisseria gonorrhoeae* is increasing globally. In recent years, gonococcal strains with resistance to the extended-spectrum cephalosporin (ESC) ceftriaxone have been reported from many countries (*1*). In South America, 7 ceftriaxone-resistant strains (MICs >0.25 μg/mL) were reported from Brazil in 2007; however, these isolates have not been characterized (*2*). Emergence of cephalosporin-resistant gonorrhea would substantially limit treatment options and represent a major public health concern. We report an *N. gonorrhoeae* isolate in Argentina that was resistant to ceftriaxone and cefixime.

In September 2014, a 19-year-old heterosexual man with no underlying disease was admitted to a hospital emergency department in Rio Negro, Argentina. Physical examination showed a purulent urethral discharge. He reported having had unprotected insertive vaginal sex with multiple partners in the past few months. The patient denied recent travel outside Argentina. He had a history of gonococcal urethritis (March 2014), which was treated with a single dose (500 mg) of ceftriaxone; a gonococcal isolate obtained at that time was not sent to a reference laboratory as part of the Argentinian Gonococcal Antimicrobial Susceptibility Surveillance Program for determination of the antimicrobial susceptibility profile.

At the time of admission, we obtained and cultured a urethral swab specimen and identified an isolate as *N. gonorrhoeae* by using conventional methods (*3*). The isolate was sent to a reference laboratory, and its identity was confirmed by using the Phadebact GC Monoclonal Test (MKL Diagnostic AB, Sollentuna, Sweden) and matrix-assisted laser desorption/ionization time-of-flight mass spectrometry (Bruker Daltonik GmbH, Bremen, Germany). The patient was given a single dose (500 mg) of ceftriaxone. Five days later, the patient returned for clinical evaluation, and symptoms had resolved. Culture for test of cure was not performed.

We determined MICs of the isolate for penicillin, cefixime, ceftriaxone, tetracycline, ciprofloxacin, and azithromycin by using the agar dilution method according to the standard of the Clinical and Laboratory Standards Institute (*4*). Results were interpreted in accordance with breakpoints of this standard, except for azithromycin, for which we applied breakpoints of the European Committee on Antimicrobial Susceptibility Testing (http://www.eucast.org/).

This isolate was resistant to ceftriaxone and cefixime (MIC 0.5 μg/mL), penicillin (8 μg/mL), tetracycline (8 μg/mL), and azithromycin (1 μg/mL). However, the isolate was susceptible to ciprofloxacin (0.03 μg/mL). We obtained a negative result for β-lactamase by using the chromogenic nitrocefin disk assay (Becton Dickinson, Franklin Lakes, NJ, USA).

Resistance determinants involved in ceftriaxone resistance were amplified by PCR and sequenced as reported (*5*,*6*). We than analyzed full-length *penA* and *pilQ* gene sequences and other genetic determinants, including *mtrR*, *penB*, and *ponA* genes. We purified and sequenced PCR products by using an ABI 3500 Genetic Analyzer (Applied Biosystems, Foster City, CA, USA) and performed molecular epidemiologic characterization by using *N. gonorrhoeae* multiantigen sequence typing (http://www.ng-mast.net).

Sequence analysis of the *penA* gene, which encodes penicillin-binding protein 2 (PBP2), identified a nonmosaic PBP2 IX allele. Analysis of the *mtrR* gene and its promoter identified a single nucleotide (A) deletion in the inverted repeat of the promoter region, and a single amino acid substitution at position H105 (H→Y). Amino acid substitutions in the *porB1b* gene were found at positions G120 (G→K) and A121 (A→D). The *ponA* gene, which encodes PBP1, had an amino acid substitution at position L421 (L→P), and full-length PilQ amino acid sequence had sequence type VI. The isolate was assigned to serogroup PorB1b (WII/III), and *N. gonorrhoeae* multiantigen sequence typing showed that it had novel sequence type ST13064 (*porB*-7592 and *tbpB*-33).

Mosaic PBP2 alleles have been strongly associated with decreased susceptibility or resistance to ESCs (*7*). However, the isolate we obtained had a nonmosaic PBP IX allele, which contains the P551L substitution that has been associated with increased MICs for ESCs (*8*). Association of the nonmosaic PBP IX allele, most likely with *mtrR*, *penB*, and *ponA* gene mutations, might be involved in resistance to ESC (*9*). Although Whiley et al. (*6*) did not report an association between sequence type VI and resistance to ESCs, the contribution of this sequence type to ESC resistance requires additional studies.

Isolates with decreased susceptibility and resistance to ESC have now emerged in Argentina (*10*), increasing from 1.1% in 2011 to 5.6% in 2014 (Argentinian Gonococcal Antimicrobial Susceptibility Surveillance Program, unpub. data). Treatment failures and isolates with reduced susceptibilities and resistance to ESC have been reported (*9*). However, in Argentina, syndromic management of gonorrhea has resulted in suboptimal diagnosis and lack of specimens to culture to distinguish between treatment failure and reinfection. Syndromic management represents a major problem that not only compromises surveillance program but also increases selective pressure and facilitates development of drug resistance. Accordingly, surveillance should be strengthened to support detection and verification of asymptomatic infections and treatment failures, identify communities at high risk, and trace sexual contacts. These efforts should be used in public health responses to mitigate emergence and spread of ESC-resistant gonococci.
